# Efficacy of Mobile Apps to Support the Care of Patients With Diabetes Mellitus: A Systematic Review and Meta-Analysis of Randomized Controlled Trials

**DOI:** 10.2196/mhealth.6309

**Published:** 2017-03-01

**Authors:** Bráulio Cezar Bonoto, Vânia Eloisa de Araújo, Isabella Piassi Godói, Lívia Lovato Pires de Lemos, Brian Godman, Marion Bennie, Leonardo Mauricio Diniz, Augusto Afonso Guerra Junior

**Affiliations:** ^1^ Post Graduate Program in Medicines and Pharmaceutical Assistance Department of Social Pharmacy Federal University of Minas Gerais Belo Horizonte Brazil; ^2^ Institute of Biological Sciences and Health Faculty of Odontology Pontifícia Universidade Católica de Minas Gerais Belo Horizonte Brazil; ^3^ SUS Collaborating Centre for Technology Assessment and Excellence in Health Department of Social Pharmacy Federal University of Minas Gerais Belo Horizonte Brazil; ^4^ Post Graduate Program in Public Health Department Preventive and Social Medicine Federal University of Minas Gerais Belo Horizonte Brazil; ^5^ Institute of Pharmacy and Biomedical Sciences University of Strathclyde Glasgow Glasgow United Kingdom; ^6^ Division of Clinical Pharmaclogy Karolinska Institutet Karolinska University Hospital Stockholm Sweden; ^7^ Department of Clinical Medicine Faculty of Medicine Federal University of Minas Gerais Belo Horizonte Brazil

**Keywords:** diabetes mellitus, self-care, mobile applications, telemedicine

## Abstract

**Background:**

Diabetes Mellitus (DM) is a chronic disease that is considered a global public health problem. Education and self-monitoring by diabetic patients help to optimize and make possible a satisfactory metabolic control enabling improved management and reduced morbidity and mortality. The global growth in the use of mobile phones makes them a powerful platform to help provide tailored health, delivered conveniently to patients through health apps.

**Objective:**

The aim of our study was to evaluate the efficacy of mobile apps through a systematic review and meta-analysis to assist DM patients in treatment.

**Methods:**

We conducted searches in the electronic databases MEDLINE (Pubmed), Cochrane Register of Controlled Trials (CENTRAL), and LILACS (Latin American and Caribbean Health Sciences Literature), including manual search in references of publications that included systematic reviews, specialized journals, and gray literature. We considered eligible randomized controlled trials (RCTs) conducted after 2008 with participants of all ages, patients with DM, and users of apps to help manage the disease. The meta-analysis of glycated hemoglobin (HbA1c) was performed in Review Manager software version 5.3.

**Results:**

The literature search identified 1236 publications. Of these, 13 studies were included that evaluated 1263 patients. In 6 RCTs, there were a statistical significant reduction (*P*<.05) of HbA1c at the end of studies in the intervention group. The HbA1c data were evaluated by meta-analysis with the following results (mean difference, MD −0.44; CI: −0.59 to −0.29; *P*<.001; I²=32%).The evaluation favored the treatment in patients who used apps without significant heterogeneity.

**Conclusions:**

The use of apps by diabetic patients could help improve the control of HbA1c. In addition, the apps seem to strengthen the perception of self-care by contributing better information and health education to patients. Patients also become more self-confident to deal with their diabetes, mainly by reducing their fear of not knowing how to deal with potential hypoglycemic episodes that may occur.

## Introduction

Diabetes Mellitus (DM) is a chronic disease that is considered a global public health problem which results in clinical, social, economic, and quality of life impacts for patients, leading to increased morbidity and mortality [1]. Complications of diabetes including cardiovascular diseases are the leading causes of death globally and are responsible for 50-80% of diabetes deaths [2]. In 2014, the global prevalence of diabetes was estimated at 9% among adults aged 18 years and older [3]. This is increasing with incidence data demonstrating an overall growth in diabetes, particularly among developing countries [4]. There are several factors associated with the rising incidence including lifestyle and diet changes. There is evidence that a large proportion of cases and complications of diabetes may be prevented by changes in lifestyle [5]. Additionally, treatment compliance by patients including control of blood pressure, a leading cause of death in patients with diabetes, is a major concern across countries [6-10].

Education and self-monitoring by diabetes patients helps to optimize and make possible satisfactory metabolic control enabling improved management and reduced morbidity and mortality [11-12]. Self-monitoring of glucose levels is also recommended for patients at risk of developing type 2 diabetes, characterizing it as an important tool for the promotion of health. In the process of encouraging patients to improve metabolic control, the importance of self-monitoring of blood glucose is one of the main strategies to assist themselves, especially those with type 1 diabetes. This highlights the importance of developing technologies to facilitate and optimize self-care, especially in the achievement of therapeutic goals for diabetic patients [11-12]. Published studies have already begun to discuss the potential of mobile apps and tablets with improving symptom management in patients with chronic diseases [13-16].

Global growth in the use of mobile phones makes them a powerful platform to help provide tailored health, delivered conveniently to patients. Several studies have documented the efficacy, challenges, and potential of mobile phones to improve health indicators in diabetes [17-24]. Mobile phones are developing rapidly mainly with regard to information processing, design, and features. These devices, called smartphones, have evolved from the ability to just make phone calls to multiple functions by combining resources on personal computers through software (apps) run by operating systems. Nowadays, the number of smartphone users is higher than traditional mobile phone users. Mobile phones allow users to install, configure, and access specialized apps on their devices [25].

Many types of apps have been developed and are available to users on the Internet such as games, entertainment, productivity, and aspects of health. Apps that contribute to health stand out in this context. In 2015, there were an estimated 500 million smartphone users in the world using apps that contributed to health care [26]. It is projected that there will continue to be a significant growth in the use of health apps, for example, by 2018 it is estimated that half of 1.7 billion “smartphone” and “tablet” users worldwide will download and use health and well-being apps [24,25].

In 2014, the Flurry platform studied app users of the health and well-being category from Apple Store [4]. An increase of 62% in the use of these apps was seen after 6 months of follow-up, with the health and well-being category growing 87% faster than the apps industry in general. This accelerated growth in apps suggests the need to conduct studies of efficacy, safety, and effectiveness to assess their benefits on patient care [27].

Consequently, given the importance and growth of mobile health apps and the potential advantages of this type of technology in addressing major concerns in the management of diabetic patients, it is important that the effectiveness of these technologies to support patient care need be evaluated.

## Methods

This principally involved a systematic review and meta-analysis of published studies using the Preferred Reporting Items for Systematic Reviews and Meta-Analysis (PRISMA) [28].

### Eligibility Criteria

The search period considered studies from 2008 to 2016. The rationale for adopting this criterion is based on the fact that in 2008, the main app stores (iOS, Android), that is those that dominate the market, were launched allowing users the autonomy to download and use apps in general. Prior to this time, the software was only distributed directly by suppliers and manufacturers, and the number of smartphone users was small. Consequently, the inclusion of studies prior to 2008 may introduce bias, characterized by other distribution format and use of apps [29].

We included RCTs and used the PICOS (participants, interventions, comparison, outcomes, study design) to define inclusion criteria (Textbox 1).

**Inclusion criteria.**

Population: adults or children were included that were diagnosed with DM type 1 or 2 (with or without comorbidities).

Intervention: mobile health apps that users input data, receive feedbacks, connect with health professionals or learn about diabetes.

- Control or comparator: any comparator was acceptable (traditional control group, an alternative intervention, or a within subject pre-post design).
- Outcome measures: the outcomes considered to evaluate the effectiveness of the apps were: biochemical parameters (HbA1c, blood glucose, total cholesterol, weight, high density lipoprotein cholesterol (HDL), low density lipoprotein cholesterol (LDL), triglycerides, blood pressure) and quality of life.

The exclusion criteria of the study are as follows:

Studies that just looked solely at the main function of mobile phones for transmitting health data by short message service (SMS) or by Internet as well as studies in which health apps had targeted health professionals were excluded. Nonrandomized studies, not controlled, quasi-experimental, and partial results were also excluded.

### Databases and Search Strategy

The research was performed in the electronic databases MEDLINE (Pubmed), Cochrane Register of Controlled Trials (CENTRAL), and LILACS (Latin American and Caribbean Health Sciences Literature) for published studies from 2008- 2016. A combination of the following MESH terms (Medical Subject Headings), “diabetes mellitus type 2,” “diabetes mellitus type 1,” “mobile applications,” “telemedicine,” and their respective entry terms were used in the strategy. In addition, a manual search was undertaken of references from identified publications and systematic reviews from 2008 for the following journals: Online Journal of Public Health Informatics; Journal of Medical Internet Research; BMC Public Health; Journal of Telemedicine and Telecare; Journal of Diabetes Science and Technology; and Journal of Telemedicine and eHealth, health and technology. With the purpose of expanding, the coverage of publications which included a search of the following gray literature sources was conducted: Digital Library of Theses and Dissertations of the University of São Paulo (USP), Digital Library of Theses and Dissertations of the Federal University of Minas Gerais general (UFMG), and electronic database ProQuest Dissertation & Theses. No language restriction was applied.

### Study Selection and Data Collection

To select studies, references were read in 2 phases (title or abstract and the full article) by 2 independent reviewers. Disagreements were resolved by a third reviewer.

After full reading of pertinent studies, a standardized form was designed to collect data from the selected studies by 2 independent researchers. The form was used to compile information about the duration and period of studies, participants at the beginning and end of each study, the age groups, health problems, and comorbidities. Interventions in both groups of participants, name and features of apps, countries where studies were conducted, clinical data, and other information were also collected.

### Assessment of Risk of Bias

The evaluation of risk of bias followed recommendations of Cochrane Collaboration. Each domain was classified as having a low risk of bias, high or unclear. This assessment was performed by 2 independent researchers and disagreements were resolved by consensus [30-31].

### Summary of Data and Statistical Analysis

Data collected from HbA1c could be combined in a meta-analysis using random effects model from *Review Manager (RevMan, computer program) version 5.3.* Results were presented as mean difference (MD) with 95% CI. Heterogeneity analysis with an I^2^> 40% and *P* value (chi-square test) <.10 were considered as significant heterogeneity. Sensitivity analysis was conducted to investigate the causes of heterogeneity, excluding 1 study each time and checking the changes in values of ​​I^2^ and *P*. Other outcomes were assessed as joint analysis because a few studies had provided enough data to be included in a meta-analysis. A subgroup analysis was also performed to check influence of exposure type that participants were submitted to, that is, conventional or remote access to health professionals and the number of features available in the app.

## Results

### Study Inclusion

**Figure 1:**
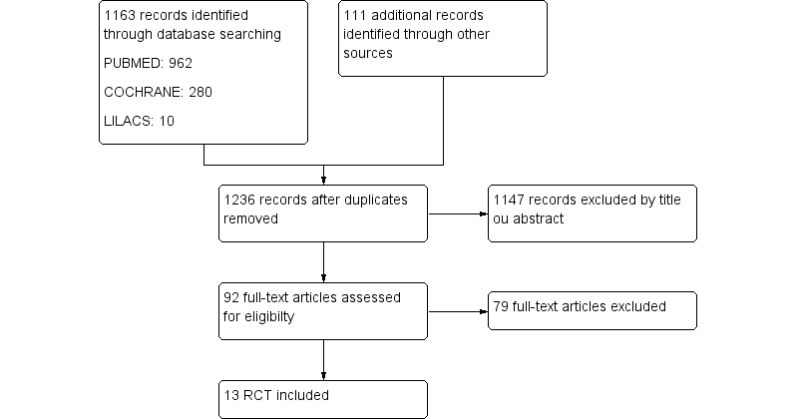
Flowchart of selection of references to systematic review.

The literature search identified 1236 publications, of which 92 were considered potentially eligible. Thirteen studies were finally included in the meta analysis [32-41]. The main reasons for the exclusions were: (1) the interventions were not apps, (2) studies were not RCT, and (3) participants were not diabetes patients (Figure 1).

### Characteristics of Studies and Participants

The included studies were performed in the United States [32,33,34], Italy [35,36], England [37], Norway [38], Germany [39], Finland [40], Australia [41], Netherlands [42], France [43] and 1 study was conducted in 3 different countries (Italy, England, and Spain) [44]. The duration of studies varied from 1 to 12 months. Of the included studies, 8 were performed in more than 1 center [32,33,35,37,38,41,43,44], whereas the remainder were performed at a single center [34,36,39,40,42]. Only 4 studies reported conflicts of interest (Table 1) [35,36,43,44].

**Table 1:** 

Study	Name (app)	Features	Country	Duration (months)
Hsu (2015) [34]	CollaboRhythm	Storage and feedback of glucose data. Graphical display of data. Storage of eating habits and physical activity. Feedback on insulin dose and calculating carbohydrate consumption. Alarms to take medicine. Telemedicine via SMS text messaging (short message service, SMS) and videoconferencing	United States	3
Drion (2015) [42]	Dbees	Storage and feedback of glucose data, carbohydrate intake, physical exercise, and medication	Netherland	3
Quinn (2014) [33]	MDMA	Storage and educational feedback of biochemical and physiological data about carbohydrate intake and medication	United States	12
Holmen (2014) [38]	Few Touch Application (FTA)	Storage and feedback of glucose data, graphical display of data, storage of eating habits and physical activity, and planning of individual goals	Norway	12
Berndt (2014) [39]	Mobil Diab (mDiab)	Storage and feedback of glucose data. Generates alerts for professionals who perform monitoring when risk is monitored	Germany	1
Nagrebetsk (2013) [37]	t+ Diabetes	Storage and graphical feedback about glucose level. Orientation aid in self-titration of oral hypoglycemic medication under the supervision of a nursing team	England	6
Kirwan (2013) [41]	Glucose Buddy	Storage and feedback of glucose data, insulin, and medication. Graphical display of data. Function to assist in diet, exercise, and planning of individual goals	Australia	9
Rossi (2013) [35]	Diabetes Interactive Diary (DID)	Storage and feedback of glucose data. Feedback on insulin dose and calculating carbohydrate consumption, telemedicine via SMS text messaging	Italy	6
Orsama (2013) [40]	Monica	Feedback on inserted biochemical parameters, graphical display of data, planning individual goals, motivational messages, and change of habits	Finland	10
Quinn (2011) [32]	MDMA	Data storage of biochemical, physiological, carbohydrate intake, and medication with educational feedback	United States	12
Castelnuovo (2011) [36]	METADIETA	Present questionnaires about weight and HbA1c, data on carbohydrate intake, connect via SMS with a nutritionist	Italy	12
Charpentier (2011) [43]	Diabeo System	Storage and feedback of glucose data. Feedback on insulin dose and calculating carbohydrate consumption. Store physical activity	France	6
Rossi (2010) [44]	Diabetes Interactive Diary (DID)	Storage and feedback of glucose data. Feedback on insulin dosage and calculating carbohydrate intake, telemedicine via SMS	Italy, England, and Spain	6

The main intervention evaluated in the studies was the use of mobile apps to assist in the monitoring of diabetes patients. In all studies, the intervention group had remote or conventional access to health professionals. Eleven different mobile apps were identified as the intervention product. The features of apps included health data storage, feedback on physiological parameters, motivational messages, function to assist with a healthy diet and exercise, functions for insulin dosage adjustment, chat and videoconferencing with health professionals, alarm for drug therapy compliance, health goals, and calculating carbohydrate intake. All participants in the control groups were subjected to standardized health treatment (Table 1).

Four studies included the percentage of participants that smoked (16% to 17%) [32,33,38,40]. Two studies also measured percentage of participants who exercised regularly [38,40]. Additionally, 34.4% [38] and 77% [40] of participants participated in physical activities. The average age of participants of these 2 studies was more than 57 years old.

The total number of participants who began studies included in this review was 1263, wherein 1068 participants took part until the end. It was found there was no association between sample loss and use of mobile apps or smartphones that would compromise outcomes. Regarding ethnicity, only 3 studies reported data. Overall, 50% or more of participants were white [32,33,37]. Education was reported in 8 studies. In 6 studies, 75% or more of the participants had, at least, completed high school [32,33,35,42-44] and 60% of sample in the intervention group were men [37-40,42]. In one study, less than 50% of the participants had completed high school [38]. Another study reported the average years of study among participants to be 11.7 years [40].

One study evaluated if the use of mobile app when compared with standard treatment, could present differences in their effectiveness based on the age of patients (≥ 55 or <55 years old). However, there were no significant differences in the outcomes measured between the 2 age groups [33]. The baseline characteristics of participants are presented in Table 2.

**Table 2:** 

Study		Sample (n)	Age in years (SD)	Gender (% men)	Participant’s disease	Disease’s duration (SD)
**Hsu (2015) [34]**					DM^a^ type 2	
	App	20	53.3 (0)	-		9.6 (0)
	Control	20	53.8 (0)	-		9.0 (0)
**Drion (2015) [42]**					DM type 1	
	App	31	33 (23)	64.5		18 (17)
	Control	32	35 (18)	62.5		15 (14)
**Quinn (2014) [33]**					DM type 2	
	App (< 55 years)	37	47.3 (6.8)	37.8		6.8 (4.5)
	App (≥ 55 years)	25	59.0 (2.9)	68.0		10.3 (5.8)
	Control (< 55 years)	29	47.4 (7.5)	62.1		8.9 (7.5)
	Control (≥ 55 years)	27	59.5 (2.8)	37.0		9.2 (6.0)	
**Holmen (2014) [38]**					DM type 2	
	App	51	58.6 (11.8)	67.0		11.2 (7.3)
	App^b^	50	57.4 (12.1)	50.0		9.6 (8.4)
	Control	50	55.9 (12.2)	40.0		9.4 (5.5)
**Berndt (2014) [39]**					DM type 1	
	App	34	12.9 (2.0)	62.0		5.0 (3.7)
	Control	34	13.2 (2.9)	56.0		5.3 (4.0)
**Nagrebetsk (2013) [37]**					DM type 2	
	App	8	56 (8.0)	71.0		3.0 (2.0)
	Control	9	60 (13.0)	71.0		2.3 (7.4)
**Kirwan (2013) [41]**					DM type 1	
	App	36	35.97 (10.67)	52.7		19.69 (9.64)
	Control	36	34.42 (10.26)	25.0		18.19 (9.77)
**Rossi (2013) [35]**					DM type 1	
	App	63	38.4 (10.3)	46.0		16.2 (10.0)
	Control	64	34.3 (10.0)	49.1		15 (8.4)
**Orsama (2013) [40]**					DM type 1	
	App	24	62.3 (6.5)	54.0		-
	Control	24	61.5 (9.1)	54.0		-
**Quinn (2011) [32]**					DM type 2	
	App	23	52.8 (8.0)	52.2		7.7 (5.6)
	App^c^	22	53.7 (8.2)	45.5		6.8 (4.9)
	App^d^	62	52.0 (8.0)	50.0		8.2 (5.3)
	Control	56	53.2 (8.4)	50.0		9.0 (7.0)
**Castelnuovo (2011) [36]**					DM type 2 or obesity	
	App	17	49 (16.5)	68.7		-
	Control	17	54 (11.7)	35.3		-
**Charpentier (2011) [43]**					DM type 1	
	App^e^	59	31.6 (12.5)	37.3		14.7 (9.1)
	App^f^	60	32.9 (11.7)	38.3		17.6 (8.9)
	Control	61	36.8 (14.1)	34.4		16.9 (10.5)
**Rossi (2010) [44]**					DM type 1	
	App	67	35.4 (9.5)	44.8		17.1 (10.3)
	Control	63	36.1 (9.4)	41.0		15.8 (10.7)

^a^DM: diabetes mellitus.

^b^Intervention is the use of the app associated with health counseling of nurses specialists in diabetes.

^c^Intervention is the use of the app and data shared with medical researchers of the study.

^d^Intervention is the use of the app and data shared with medical researchers of the study associated with quarterly reports delivered to participants from data entered.

^e^Intervention is the use of the app and access health professionals as control group.

^f^Intervention is the use of the app and access health professionals remotely.

### Risk of Bias

**Figure 2:**
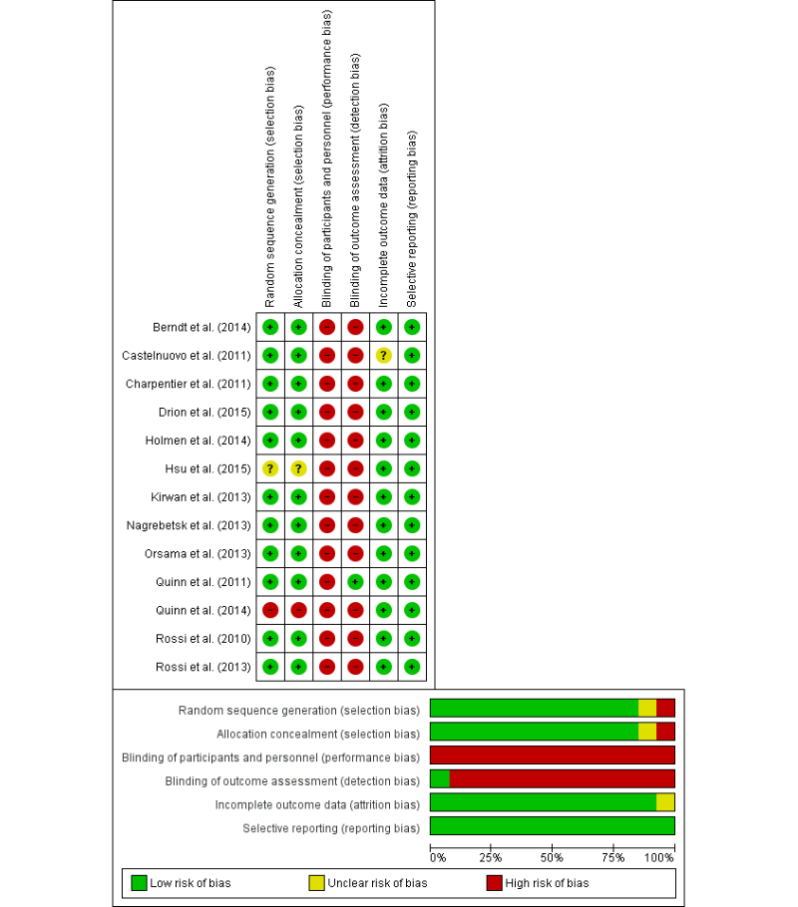
Analysis of the risk of bias.

When evaluating risk of bias, 11 out of the 13 studies presented low risk of selection bias [32,35-44] and 1 showed unclear data [34]. However, in the performance and detection categories, all studies presented high risk of bias. Only 1 study showed unclear data on incomplete outcomes [36]. All studies had low risk on selective reporting (Figure 2).

### Glycated Hemoglobin and Hypoglycemic Episodes

HbA1c was measured in 12 studies [32-35,37-44]. In 6 studies, there were statistical significance difference in the reduction of this parameter favoring the intervention within 12 months of follow-up (*P*<.03) [32-34,40,41,43].

**Figure 3:**
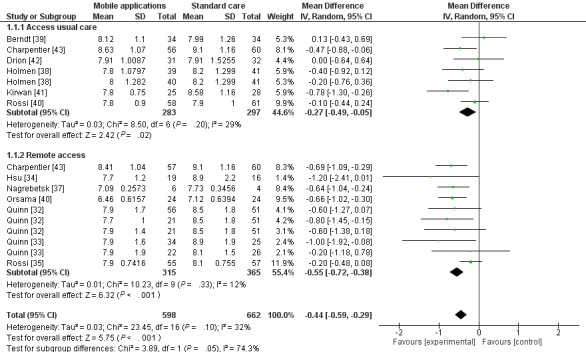
Forest-plot of glycated hemoglobin of diabetes patients who used a health app and have access physically or remotelly to health professionals.

Overall, the meta-analysis showed the effectiveness of the use of apps to control diabetes (*P*<.001), with lower heterogeneity (MD −.44; CI −0.59 to −0.29; *P*<.10; I²= 32%). The sensitivity analysis showed that excluding the study [41] in the subgroup “Access to usual care” and [35] in the subgroup “Remote Access,” there was a reduction of heterogeneity in both subgroups to zero without changing the direction of outcome (Figure 3).

Hypoglycemic episodes were reported in 5 studies [34,35,39,43,44]. In 1 study, 30 and 33 mild episodes were recorded in the intervention and control groups respectively and a serious episode in the control group [39]. In 3 studies, episodes were recorded in each group without significant difference [34,43,44]. In a third study, the intervention group had a lower relative risk (0:14; CI 0.07-0.029) of severe hypoglycemic episodes. [35]

### Subgroup Analysis

Subgroup analysis was performed to evaluate if the route of access to health professionals for monitoring diabetes, in addition to the app, affected outcomes in terms of HbA1c. In 7 studies, participants in the intervention group had access to health professionals remotely [32-35,37,40], in 5 studies, participants had access to usual care [38,39,41,42,44,] and in 1 study intervention participants had access to health professionals remotely or physically [43]. Both subgroups showed favorable results in HbA1c control (Figure 3).

The number of features available in the app of each study was also evaluated to check their impact on HbA1c. Four main features were identified in apps that contributed to achieving glycemic control. These were “storage and feedback of blood glucose data,” “function to assist in diets,” “function to aid at physical exercises practice,” and “control over dosage and adherence to drug therapy” [45].

**Figure 4:**
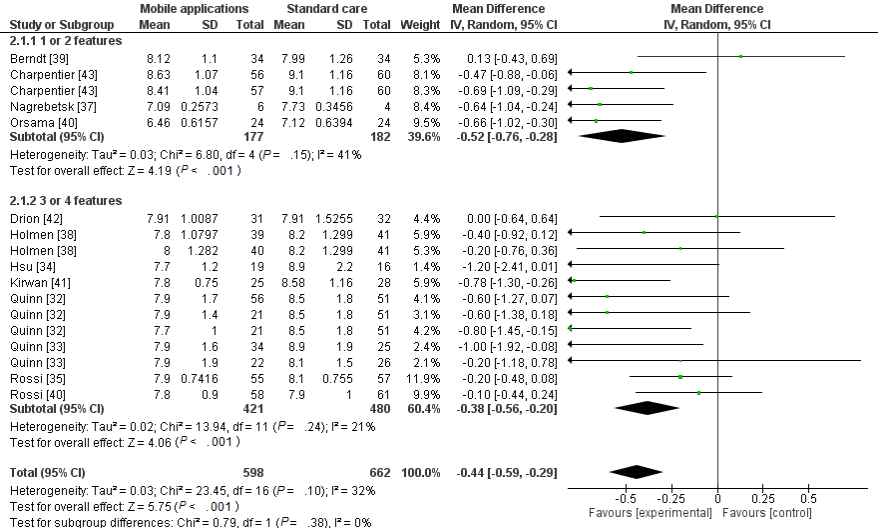
Forest plot of glycated hemoglobin of diabetes patients who used a health app according to the number of selected app features.

Subgroups were separated in order to evaluate studies where the apps provide 1 or 2 features of the 4 identified for glycemic control (*P*=.05) [37,39,40]. It was demonstrated that the subgroup with fewer features in an app had outcomes with borderline significant difference. The subgroup where apps had more than 2 functionalities generated the following results (*P*<.001) [32-35,38,41-44] (See Figure 4).

A subgroup analysis was performed to evaluate if there was a difference among different types of diabetes mellitus. Both subgroups showed favorable results of HbA1c control to intervention group compared with control group.

### Secondary Outcomes

Different secondary outcomes were evaluated in some studies. Four studies were conducted using an assessment of fasting blood glucose assessment. However, there was no significant reduction in any study [35,39,41,44]. Six studies assessed weight changes [35,36,38-40,44]. Four studies assessed changes in blood pressure [32,35,40,44] and 3 studies measured total cholesterol, high-density lipoprotein, low-density lipoprotein, and triglycerides [32,35,44]. The results were presented by a joint analysis (Table 3).

**Table 3:** Joint analysis of secondary outcomes.

Outcome	Intervention (n)	Control (n)	Mean difference (95% CI)	*P* value	I² (%)
Fasting blood glucose [35,39,41,44]	172	180	0.05 (−1.39 to 1.49)	.95	79%	
Body weight [35,38,39,44]	226	193	−0.39 (−1.43 to .66)	.47	0%
Systolic blood pressure [32,35,40,44]	221	179	0.10 (−2.36 to 2.55)	.94	0%
Diastolic blood pressure [32,35,40,44]	221	179	0.37 (−1.10 to 1.85)	.62	0%
Total cholesterol [32,35,44]	211	169	−3.44 (−12.87 to 6.00)	.48	44%
High-density lipoprotein [32,35,44]	211	169	−2.15 (−5.40 to 1.10)	.19	58%
Low-density lipoprotein [32,35,44]	211	169	1.69 (−5.67 to 9.06)	.65	26%
Triglicerides [32,35,44]	211	169	−14.67 (−33.40 to 4.06)	.12	58%	

Quality of life was assessed in 6 studies using different measuring instruments: Disease-Specific Quality-of-Life (DSQOL) [35], Diabetes Quality of Life (DQOL) [41], Diabetes Quality of Life for Youths (DQOLY) [39], and 36-Item Short-Form (SF-36) [38,42,44]. Three studies found positive and statistical significant changes in quality of life and satisfaction with treatment in the intervention group [35,39,44]. Health improvements reported by participants with the app were the perception of hyperglycemia episodes, social relationships, decreased fear of hypoglycemia, perception that the apps aid treatment, and healthier dietary habits.

## Discussion

### Principal Findings

The meta-analysis found a significant difference throughout 12 months among the intervention group in terms of better HbA1c control. However, overall there were no significant differences with respect to secondary outcomes between the groups. These results indicate relevant questions about the potential of tools for self-monitoring and self-care by patients and the role of remote access to health care professionals where there appears to be similar effectiveness with conventional access to diabetes patients.

We believe it is worth mentioning that while these results have shown significant differences compared with the control group for control of HbA1c, only 2 studies [37,40] reached values ​​considered suitable for glycemic control, which is 7%, according to the global consensus [46,47]. This demonstrates the major challenge in achieving satisfactory results in the treatment of diabetes, despite all groups of participants having shown better average results at the end of the studies.

In other studies that averaged more than 7%, the maximum average value found was 8.63% in the intervention group [43]. The United Kingdom Prospective Diabetes Study shows that every percentage point decrease in HbA1c reduces by 35% the risk of vascular complications [48]. Another study showed that HbA1c values ​​between 7.4(1.4) and 7.7(1.4) do not increase the risk for retinopathy and nephropathy, respectively, while values ​​above 9.3(1.1) and 9.6(1.2) show increased risk of development and progression of retinopathy and nephropathy, respectively [49].

Association between use of apps and remote access to health professionals demonstrated great effectiveness in controlling HbA1c. Studies in which intervention groups accessed health professionals similar to the control groups also showed significance difference in outcomes. This suggests that the use of apps by themselves may not be more effective than standard treatment. Apps have better results when they include tools of remote communication with health professionals or access them face to face.

The number of features that apps offer also appears to influence HbA1c levels. Studies in which apps had even 2 features showed borderline results between the 2 groups. Results were favorable when more than 2 features of control were available in the app, ie, more than 2 of “storage and feedback of blood glucose data,” “function to assist in diets,” “function to aid at physical exercises practice,” or “control over dosage and adherence to drug therapy.”

Studies evaluating quality of life reported that use of apps have increased the perception of knowledge by participants about their health problems. This may represent a contribution to perceived need for self-care by users [35,39,44]. These results corroborate the proposed measures of health promotion by the International Diabetes Federation [12].

The high risk of bias for blinding participants and masking interventions is followed by almost the impossibility of health professionals and patients unaware of the use of apps and smartphones in care process. However, some studies reported there is no empirical evidence to support the conclusion that problems in masking the interventions may compromise the results [50,51].

An important characteristic measured in these studies was participants’ education. It is expected that individuals with higher education have greater ability in adopting new technologies. This may be a limitation of the studies because results favoring app users might not have had the same outcomes if participants had less education. In a study conducted in Norway [38], most of the participants had education below high school, and any measured outcomes showed results with significant difference for 1 of the groups. However, the Norwegian study may not be a good reference because Nordic countries are highly digitalized societies, which is not yet a reality in a number of countries including Brazil [52].

All studies included in this systematic review were undertaken in developed countries and therefore it is necessary to measure the ability to generalize with developing countries. Access to apps requires the presence of a smartphone or tablet and Internet access for satisfactory performance. In Brazil, statistics from the Web-based statistics portal Statista suggests that by 2017, 42.5% of mobile users will be smartphone users [53]. In absolute numbers, Brazil will have nearly 170 million mobile phone users by 2018 [54], suggesting that more than one third of Brazilians will have access to a smartphone by 2018. According to the World Bank, Internet access in Brazil in 2014 reached 57.6% of the population [55], allowing the potential use of apps in health care processes.

Age may also be an influencing factor to the adoption of new technologies [56-60]. In 5 of the studies, participants had an average age under 40 years [35,41-44]. In other studies, participants had a mean age of 50 years, except 1 study with teenagers. Studies with participants with an average age of 40 years showed improvements in outcomes including HbA1c [41,43], triglycerides [44], and a relative risk reduction shield for hypoglycemic episodes [35]. However, 1 study showed no significant difference in outcomes among people under and over 55 years old in the 2 groups [34].

A last important analysis was related to a higher proportion of men (60% of sample) in the intervention group of some studies [37-40,42]. Reference showed that men were more interested in adopting new technologies, while women preferred to take opinions before use [61]. However, the included studies in this review showed men and women had comparable results.

After performing the analysis, it can be concluded that use of apps for diabetes control as an aid to treatment can be considered an effective measure, especially when patients have access to health professionals. Sustainable health systems need to invest in disease prevention and health promotion actions. Self-monitoring actions aim to raise awareness and education about the role of patients and family in managing their health problems. At the same time, smartphones with Internet access have the potential to provide data from clinical parameters measured at home that can relieve pressures on health systems directly due to improved access for those who really need to use clinics and hospitals and, indirectly, by reducing costs and increasing therapeutic effectiveness.

The results from this meta-analysis suggest that self-monitoring can be delivered by smartphones, with increasing use of smartphones by people from different socioeconomic conditions. The use of such devices can still be considered complex and potentially a barrier to access among elderly patients. However, in the medium term, population aging will include almost all in a highly connected and digitalized society.

### Conclusions

This systematic review suggests that use of apps in patients with diabetes could help improve the control of HbA1c. In addition, the apps seem to strengthen the perception of self-care by contributing better information and health education to diabetes patients. App features including “storage and feedback of blood glucose data,’ ‘assist in diet,” “help practice in physical exercise,” and “assist in control of dosage and adherence to drug therapy” as well as access to health care professionals contributes to a better glycemic control. Patients also become more self-confident to deal with their diabetes, mainly by reducing fear of not knowing how to deal with potential hypoglycemic episodes that may occur and improving their quality of life.

## Abbreviations

DM: diabetes mellitus
HbA1c: glycated hemoglobin
MD: mean difference.
RCTs: randomized controlled trials
SMS: short message service
